# First description of antimicrobial resistance in carbapenem-susceptible *Klebsiella pneumoniae* after imipenem treatment, driven by outer membrane remodeling

**DOI:** 10.1186/s12866-020-01898-1

**Published:** 2020-07-20

**Authors:** Xuebin Tian, Qiongdan Wang, Laura Perlaza-Jiménez, Xiangkuo Zheng, Yajie Zhao, Vijay Dhanasekaran, Renchi Fang, Jiahui Li, Chong Wang, Haiyang Liu, Trevor Lithgow, Jianming Cao, Tieli Zhou

**Affiliations:** 1 Department of Clinical Laboratory, The First Affiliated Hospital of Wenzhou Medical University, Wenzhou, Zhejiang Province China; 2 School of Laboratory Medicine and Life Sciences, Wenzhou Medical University, Wenzhou, Zhejiang Province China; 3 Infection and Immunity Program, Biomedicine Discovery Institute and Department of Microbiology, Monash University, Melbourne, VIC Australia

**Keywords:** *Klebsiella pneumoniae*, Outer membrane porin, Imipenem treatment, Carbapenem resistance, Antimicrobial resistance

## Abstract

**Background:**

The emergence of carbapenem-resistant *Klebsiella pneumoniae* (CRKP) poses a looming threat to human health. Although there are numerous studies regarding porin alteration in association with the production of ESBLs and/or AmpC β-lactamase, a systematic study on the treatment-emergence of porins alteration in antibiotic resistance does not yet exist. The aim of this study was to investigate the underlying mechanism of resistance of *K. pneumoniae* during carbapenem treatment.

**Results:**

Here, we report three strains (FK-2624, FK-2723 and FK-2820) isolated from one patient before and after imipenem treatment during hospitalization. Antibiotic susceptibility testing indicated that that the first isolate, FK-2624, was susceptible to almost all tested antimicrobials, being resistant only to fosfomycin. The subsequent isolates FK-2723 and FK-2820 were multidrug resistant (MDR). After imipenem therapy, FK-2820 was found to be carbapenem-resistant. PCR and Genome Sequencing analysis indicated that *oqxA,* and *fosA5*, were identified in all three strains. In addition, FK-2624 also harbored *bla*_SHV__-__187_ and *bla*_TEM-116_. The *bla*_SHV__-__187_ and *bla*_TEM-116_ genes were not detected in FK-2723 and FK-2820. *bla*_DHA__-1_, *qnrB4*, *aac (6′)-IIc, and bla*_SHV__-__12_, *EreA2, CatA2, SulI, and tetD*, were identified in both FK-2723 and FK-2820. Moreover, the genes *bla*_DHA_-1, *qnrB4*, *aac (6′)-IIc* were co-harbored on a plasmid. Of the virulence factors found in this study, *ybtA, ICEKp6, mrkD, entB, iroN, rmpA2–6, wzi16* and capsular serotype K57 were found in the three isolates. The results of pairwise comparisons, multi-locus sequencing typing (MLST) and pulsed-field gel electrophoresis (PFGE) revealed high homology among the isolates. Sodium dodecyl sulfate polyacrylamide gel electrophoresis (SDS-PAGE) results showed that isolate FK-2820 lacked OmpK36, with genome sequence data validating that there was a premature stop codon in the *ompK36* gene and real-time RT-PCR suggesting high turnover of the *ompK36* non-sense transcript in FK-2820, with the steady-state mRNA level 0.007 relative to the initial isolate.

**Conclusion:**

This study in China highlight that the alteration of outer membrane porins due to the 14-day use of imipenem play a potential role in leading to clinical presentation of carbapenem-resistance. This is the first description of increased resistance developing from a carbapenem-susceptible *K. pneumoniae* with imipenem treatment driven by outer membrane remodeling.

## Background

*Klebsiella pneumoniae* is a serious hospital-acquired pathogen causing infections such as urinary tract infections, pneumonia and bloodstream infections [1, 2]. The abuse of expanded-spectrum cephalosporins for the remedy of these organisms has contributed to the appearance of extended-spectrum β-lactamases (ESBLs) and AmpC-producing *K. pneumoniae.* Carbapenems had been considered as the last resort for the treatment of infections caused by multidrug-resistant (MDR) isolates, due to their broadest antibacterial spectrum compared to other β-lactams. However, carbapenem-resistant *K. pneumoniae* (CRKP) have increasingly emerged under antibacterial drug selection pressure. The acquisition of plasmid-borne genes encoding carbapenemases is the mainly mechanism of CRKP since the first isolation of CRKP in America in 1996 [3]. Recently, it has been shown that carbapenem-resistance can also occur in the absence of carbapenemases, resulting instead from porin alteration so long as it occurs in association with the production of ESBLs and/or AmpC β-lactamase [4]. The porin OmpK36 is a nonspecific, passive diffusion pore in the outer membrane of *K. pneumoniae*, belonging to the OmpC porin group [5]. Several reports now support the contention that loss of Ompk36, coupled with ESBL and/or AmpC production, can contribute to CRKP phenotypes [6, 7]*.* The current study focused on the acquisition of carbapenem resistance determinants in *K. pneumoniae* isolated from one inpatient, with emphasis on determining appropriate antimicrobial course of treatment.

## Results

### Clinical characteristics of the patient

**Fig. 1:**
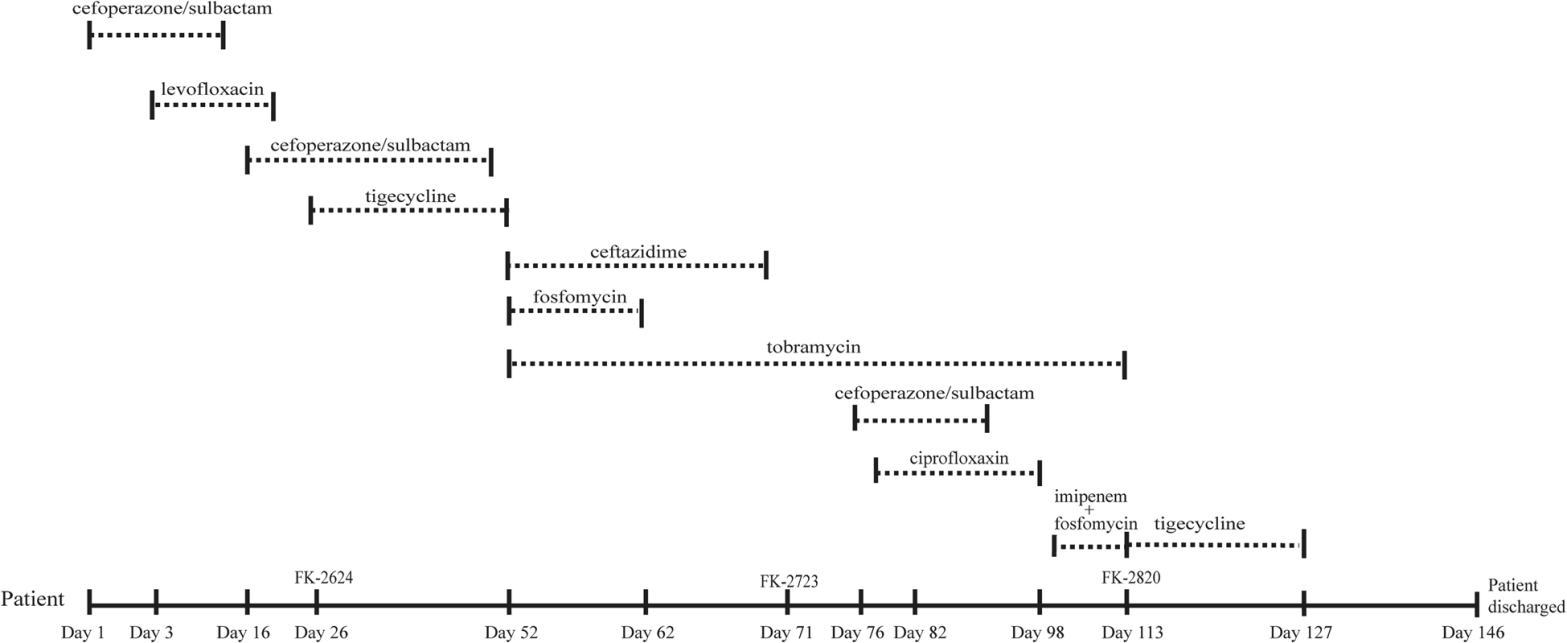
Treatment and diagnostic timeline. Timeline representing the days at which the isolates were obtained from patient and the respective interventions that were performed

This case was a 62-year-old male patient admitted to the department of neurosurgery in the First Affiliated Hospital of Wenzhou Medical University in October 2015 with headache. A diagnosis of cerebral aneurysm with subarachnoid hemorrhage was made, with a secondary pulmonary infection occurring after surgery. Antimicrobial treatment was started with 2 g of cefoperazone/sulbactam every 8 h (q8h), 0.5 g levofloxacin two times a day (bid.). On day 26, the fosfomycin-resistant *K. pneumoniae* FK-2624 was isolated from a sputum sample (Table 1). After that, due to pneumonia, the patient was switched to tigecycline via vein infusion (ivgtt.) with a loading dose of 100 mg. On Day 50, chest CT suggested atelectasis, which required a tracheotomy. On day 52, *Pseudomonas aeruginosa* was detected in purulent sputum, and the patient was switched to fosfomycin with an ivgtt. Loading dose of 8 g q12h, and tobramycin with an ivgtt. Loading dose of 80 mg q12h due to susceptible to tobramycin (Table S3). On day 71, isolate FK-2723 was identified from the patient’s sputum sample and microbiological characterization found FK-2723 to be resistant to ceftriaxone, ceftazidime, levofloxacin, ciprofloxaxin, tobramycin, gentamicin and fosfomycin. This isolate was found to be susceptible to carbapenems, colistin and tigecycline. On day 99, the patient was started on a 14-day course of combination treatment with imipenem and fosfomycin. On Day 113, FK-2820 was isolated and shown to be carbapenem-resistant but was susceptible to tigecycline (Table 1). Tigecycline treatment was initiated on Day 113 and after another 33 days hospitalization, the patient was transferred to a superior hospital in poor health, graded 6 by Glasgow coma scale (GCS) criterion (Fig. 1).

**Table 1:** Phenotypic detection, distribution of resistance genes, MICs and virulence factors of antimicrobial agents among the *K. pneumoniae* isolates

Isolates codes	MHT^1**)**^	EDTA	Resistance genes profiles	MIC (μg/ml)														Relative expression of		Virulence factors
				CRO^**2)**^	CAZ	CTX	IPM	MEM	ETP	LEV	CIP	TOB	GEN	AMK	FOS	COL	TGC	***ompK35***	***ompK36***	
**FK-2624**	–	–	*bla*_SHV-187,_*oqxA*. *fosA5, bla*_TEM-116_	0.5	1	0.5	0.125	0.03	0.03	0.5	0.5	0.5	1	1	256	0.25	2	0.93	12.67	K57, *ybtA, ICEKp6, mrkD, entB, iroN, rmpA2–6, wzi16, rmpA-2(KpVP-1)*
**FK-2723**	–	–	*bla*_SHV-12_, *bla*_DHA-1_*qnrB4*, *oqxA, aac(6′)–IIc*, *fosA5, EreA2, CatA2, SulI, TetD*	8	> 64	16	1	0.25	0.5	8	4	128	32	4	> 1024	1	1	1.61	1.39	K57, *ybtA, ICEKp6, mrkD, entB, iroN, rmpA2–6, wzi16*
**FK-2820**	–	–	*bla*_SHV-12_, *bla*_DHA-1_*qnrB4*, *oqxA, aac(6′)–IIc*, *fosA5, EreA2, CatA2, SulI, TetD*	8	> 64	64	8	1	> 16	8	4	128	64	16	> 1024	1	1	1.44	0.093	K57, *ybtA, ICEKp6, mrkD, entB, iroN, rmpA2–6, wzi16*

**Note:**^1)^*MHT* Modified Hodge test, *EDTA* EDTA synergy test

^2)^*CRO* Ceftriaxone, *CAZ* Ceftazidime, *CTX* Cefotaxime, *IPM* Imipenem, *MEM* Meropenem, *ETP* Ertapenem, *LEV* Levofloxacin, *CIP* Ciprofloxacin, *TOB* Tobramycin, *GEN* Gentamicin, *AMK* Amikacin, *FOS* Fosfomycin, *COL* Colistin, *TGC* Tigecycline

### Antimicrobial susceptibility testing, characterization of resistance genes and virulence factors

The antibiotic resistance profiles, determined microbiologically, are shown in Table 1. The initial two isolates were carbapenem-sensitive. In the case of FK-2820, MICs for imipenem and ertapenem were 8 and 16 μg/mL, respectively. Although FK-2820 remained clinically sensitive to meropenem, the MIC of meropenem had increased four times, from 0.25 μg/mL to 1 μg/mL.

Initially, a phenotypic method was used for the detection of carbapenemase production. The mCIM analysis and EDTA synergy test are biochemical assay for carbapenemase activity, and the results were negative for FK-2820, suggesting that it does not express a genuine carbapenemase (Table 1). In order to independently test this result, to determine whether or not FK-2820 has a gene encoding a carbapenemase that would explain the observed carbapenem-resistance, whole genome sequencing (WGS) was undertaken directed at the detection of mechanism in FK-2820 that distinguish it from FK-2723.

WGS was used to search for the presence of genes encoding virulence factors. Of the virulence factors found in this study, *ybtA, ICEKp6, mrkD, entB, iroN, rmpA2–6, wzi16* and capsular serotype K57 were found in the three isolates (Table 1). These virulence genes are associated with high-affinity iron chelators or siderophores, iron uptake, biofilm formation and infection. Additionally, FK-2624 carries *rmpA-2* which is not found in the subsequent isolates and is commonly carried on virulence plasmids such as *KpVP-1* [8]. This raised the hypothesis that the initial isolate FK-2624 carried a virulence plasmid that had been lost from the subsequent isolates.

WGS was used to search for the presence of resistance genes, and these resistance markers were confirmed by PCR amplification using diagnostic primers, followed by DNA sequencing. Two resistance markers: *oqxA,* and *fosA5*, were identified in all three strains. In addition to these markers, FK-2624 also harbored *bla*_SHV__-187_ and *bla*_TEM-116_ (Table 1). The *bla*_SHV__-187_ and *bla*_TEM-116_ genes were not detected in the WGS data from FK-2723 and FK-2820. An initial hypothesis was that, like the virulence gene *rmpA-2*, they may have been carried on a plasmid found only in FK-2624.

The following antibiotic resistance markers: *bla*_DHA-1_, *bla*_SHV__-__12_, *qnrB4*, *aac (6′)-IIc, and EreA2, CatA2, SulI, and tetD*, were identified in both FK-2723 and FK-2820. Sequence analysis suggested that the genes *bla*_DHA__-1_, *qnrB4*, *aac (6′)-IIc* were co-harbored on a plasmid, and results of BLASTn analysis suggested that this plasmid displays a high level of sequence identity to plasmid pR47–309 (Genbank accession CP040697.1).

### Substractive characterisation of the *K. pneumoniae* isolate genomes

**Fig. 2:**
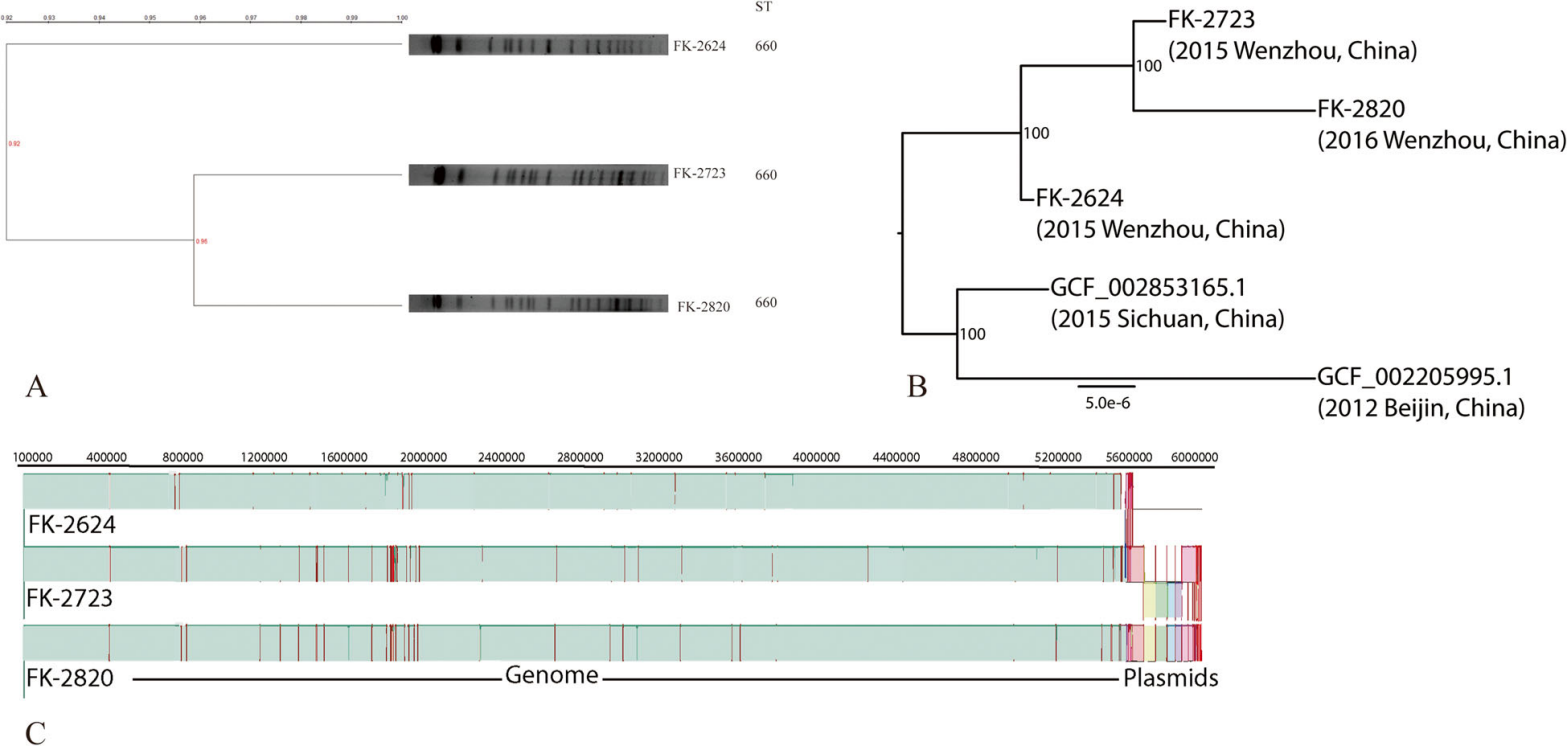
PFGE analysis and MLST of 3 *K. pneumoniae* isolates. **a** Relatedness was analyzed using Quantity One software (Bio-Rad Laboratories, USA). The phylogenetic tree was generated using UPGMA clustering. A genetic similarity index scale is indicated by the vertical line. **b** Phylogeny of the three isolates and two other ST660 genomes from NCBI. Labels in the nodes are bootstrap support. The scale represents the number of substitutions per site. **c** MAUVE alignment of the three genome sequence datasets. SNPs are represented by vertical red lines, colored boxes at the far end of the alignment correspond to putative plasmids acquired or lost from each of the subsequent isolated strains

MLST analysis showed that the three strains belong to ST660, and are closely related (Fig. 2, Fig. S2, Table S4). An initial phylogenetic analysis of those few ST660 *K. pneumoniae* for which sequence data is available (Fig. 2b) showed that these closely related genomes from the NCBI database, collected in Beijing and in Sichuan (in 2012 and in 2015, respectively), are classified as *Klebsiella pneumoniae subsp ozaena.* The pairwise sequence identity was calculated to be > 98% across the genomes of the three strains collected in this study (Table S2), with no more than 410 single polymorphisms in the core genome (Table S2).

Alignment of the sequence data from the three isolates (Fig. 2c, Table S5) shows a short region of discrepancy in the length of the genome and dissimilarity of sequence characteristics, consistent with the presence of plasmids. FK-2624 has ~ 160 kb of distinguishing DNA, while FK-2723 has ~ 380 kb and FK-2820 has ~ 400 kb of DNA that might correspond to plasmids (Table S5). Differences in GC-content of those dissimilar regions compare to the rest of the alignment are consistent with this. These physical findings for the genome support the earlier observations concerning the differences in virulence and resistance genes in the three strains, and the putative plasmid in FK-2624 encompasses the genes encoding the *TEM-116* beta-lactamase (*bla*_TEM-116_) and the virulence factors *iro-1* and *rmpA-2* specific to that isolate (Table 1).

Further analysis of the WGS data for FK-2327 and FK-2820 showed that the putative plasmid DNA harbors the genes *qnrB4, ereA2, catA2, sulI, tetD*. These genes confer resistance to aminoglycosides; fluoroquinolones, macrolides, phenicols, sulphonamides, and tetracyclines, respectively. Additionally, FK-2723 and FK-2820 plasmids carry the gene *bla*_DHA-1_ encoding a beta-lactamase.

### OMP analysis

**Fig. 3:**
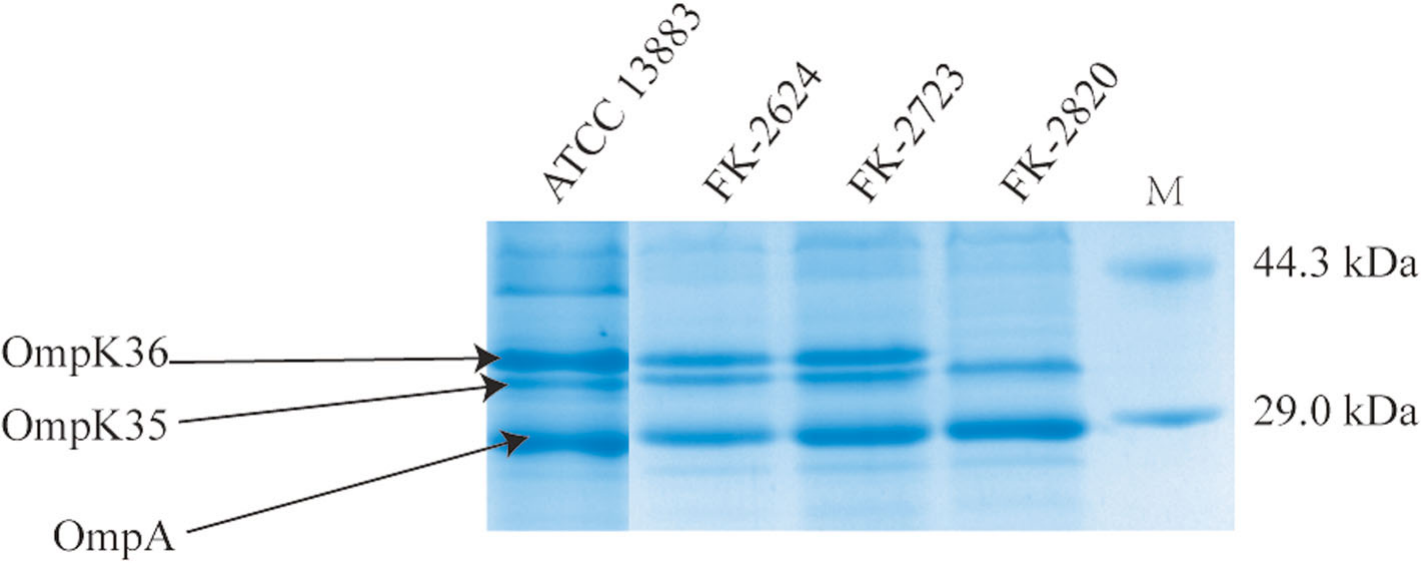
OMP analysis from the *K. pneumoniae strains*. Extracts prepared from the three isolates (FK-2624, FK-2723 and FK-2820) and the control *K. pneumoniae* ATCC 13883 were subject to SDS-PAGE and staining with Coomassie blue. Lanes M, protein mass marker. ATCC 13883, an isolate with known expression of both OmpK35 and OmpK36

Characterization of the outer membrane protein profile by SDS-PAGE showed that isolate FK-2820 did not express a full complement of porins (Fig. 3, Fig. S1). The relative expression levels for two of the three major protein species, namely OmpA and OmpK35 are similar for the three patient isolates and a reference strain of *K. pneumoniae* ATCC 13883 (Fig. 3, Fig. S1). On this gel system, the major porin OmpK36 migrates as a band of ~ 35 kDa [6] and, while it was evident in the other strains tested as the major protein species, the protein-based analysis showed that OmpK36 expression is not observed for FK-2820.

To understand the genetic basis for this loss of OmpK36 expression, we undertook direct sequencing of the *ompK36* gene. PCR-based evaluation was confirmed by preliminary analysis of the WGS data, revealing a frameshift mutation caused by a substantial deletion of 239 nucleotides, changing the reading frame and truncating the sequence of OmpK36 in FK-2820. This partial protein in FK-2820 is incapable to assemble a β-barrel, and would be subject to quality control proteases such as DegP and BepA known to degrade other defective β-barrels [9, 10].

While the transcriptome evaluations did not mirror the protein-based evaluation, they did yield consistent results. Compared with the initial isolate *K. pneumoniae* FK-2624, expression of *ompK35* was similar in FK-2723 and FK-2820 (1.70 and 1.54, respectively). In the case of *ompK36* there was a ten-fold decrease in the steady-state level of the transcript that did not change the protein steady-state level in FK-2723 (Fig. 3, Fig. S1), but the decrease in *ompK36* transcript levels observed in FK-2820 was substantial and significant (0.007 relative to the initial isolate, *P* < 0.05) (Table 1), consistent with the increased decay of at least some transcripts carrying premature stop codons [11].

## Discussion

Bacterial antimicrobial resistance has emerged as one of the main concerns of public health and constitutes a major, global challenge of the near future [12]. As a key example, the emergence and global spread of CRKP poses clinical and therapeutic challenges, since they exhibit resistance to the majority of commonly used antibiotics [13]. Carbapenems are the class of β-lactam antibiotics with strong activity against many Gram-positive and Gram-negative species, making them key treatment options in infections resistant to other drugs. However, since the first report of KPC-2 strains of *K. pneumoniae* [14], various carbapenemase-producing isolates are being identified across the globe [15]. In addition to the carriage of carbapenemases such as KPC-2, porin alteration can also play a role in establishing CRKP phenotypes in strains which produce ESBLs or AmpC β-lactamases [4]. In the current study, resistance was monitored and characterized in three strains isolated from one patient, with the aim of our study being to investigate the mechanism of treatment-emergent carbapenem-resistance.

FK-2624 was isolated from the patient on day 26 and shown to be susceptible to most commonly used antimicrobial agents except fosfomycin. By contrast, the MDR strain FK-2723 isolated on day 71 only exhibited susceptibility to carbapenems, colistin and tigecycline. After imipenem treatment for 14 days, the isolate FK-2820 was found to be carbapenem-resistant. WGS and molecular epidemiology analysis revealed homology among FK-2624, FK-2723 and FK-2820, and the difference in resistance patterns between FK-2624 and the other two strains was due to acquisition of plasmids that conferred certain resistance phenotypes to FK-2723 and FK-2820. All of tested strains harbored the gene *fosA5*, which confers high level resistance to fosfomycin [16]. In addition to these core genes, three resistance genes were inferred to be plasmid-borne: *bla*_DHA-1_, *qnrB4* and *aac (6′)-IIc*, and the coexistence of multiple resistant genes in these two isolates may contribute to high-level resistance to the majority of clinically available antimicrobial agents.

Moreover, *K. pneumoniae* strains could produce different virulence factors, such as fimbrial adhesins and siderophores, which are important in the colonization and development of the infection. To date, the delineation of hypervirulent *K. pneumoniae* (hvKp) virulence genes remains incomplete, and it remains unclear which genes are needed for maximal virulence [17]. We hold that the good markers should be critical factors in conferring the hypervirulent phenotype. If such markers are lost, then the phenotype will no longer be hypervirulent. A recent study demonstrated that *iroB*, *iucA*, *peg-344*, *rmpA* and *rmpA2* were the most accurate molecular markers for defining hvKp [18]. In current study, none of the three strains carried the hypervirulent markers, but the virulence genes *ybtA*, *mrkD*, *entB* and *iroN* in the MDR background of these *K. pneumoniae* isolates may further exacerbate infections caused by these bacteria and hamper treatment. *K. pneumoniae* ST660 remains uncommon in clinical infections, with only two ST660 strains in the publicly accessible database (http://bigsdb.pasteur.fr/). The emergence of this further case warrants further surveillance for ST660.

Our sequencing results showed the acquisition of mutations leading to a reading-frame change and early termination of translation in the *ompK36* coding sequence. The truncated porins were not detected, which is expected given that only a complete polypeptide can form the mature β-barrel structure [9]. Our study provided interesting data on the mRNA responses of *K. pneumoniae* to carbapenem antibiotics. Expression of *ompK36* was substantially decreased in FK-2820, which was consistent with the absence of the corresponding protein band in the SDS-PAGE analysis. However, the difference between FK-2624 and FK-2723 expression of *ompK* 36 were not reflected in the protein steady-state levels. In other words, transcriptional responses in *K. pneumoniae* were not predictive for protein steady-state levels. Relatively little is known about OMP regulation in *Klebsiella*, but expression of *ompK36* may be subject to negative regulation through factors such as *micF* and *ompR* [19]. These, in turn, could impact on susceptibility to carbapenems and the regulatory cascades that might buffer these effects would be worthy of future investigation. Our results demonstrated the alteration of OmpK36 alone may confer a high level of resistance to ertapenem, consistent with previous report [20]. Thus, further studies are needed to understand the pathways of regulation for resistance-related genes in *K. pneumoniae* during antibiotic pressure. A recent study revealed that a 13-day in vitro carbapenem resistance induction showed loss of OmpK 36 in mutant isolate [21]. In current study, our data showed that a 14-day treatment in vivo of MDR *K. pneumoniae* infection was not particularly effective in clearing MDR *K. pneumoniae* the sputum. Furthermore, treatment with imipenem could lead to a premature stop codon of the *ompK36* gene in the carbapenem-susceptible MDR *K. pneumoniae*, leading to the development of carbapenem resistance. The report first described the remodeling of outer membrane porin from carbapenem- susceptible MDR *K. pneumoniae* to carbapenem-resistant *K. pneumoniae* from a genomic perspective in vivo. This will give more inspiration to clinical anti-infective treatment. To our knowledge, this study in China is the first description of a connection between imipenem treatment and alteration of OmpK36 in *K. pneumoniae*.

## Conclusions

This study manifested a link between imipenem treatment of patients infected by MDR *K. pneumoniae,* and the sequential alteration of porin expression in isolates of *K. pneumoniae* collected during the 14 days treatment. The evidences the need for rational use of antibiotics during patient admission, for the understanding of the impact of treatment-mediated changes to drug-resistance phenotypes, and for effective surveillance to provide guidance on the utility of antimicrobial agents to treat *K. pneumoniae* infections.

## Methods

### Bacterial isolates

Between October 2015 to February 2016, *K. pneumoniae* strains FK2624, FK-2723 and FK-2820 were isolated from the sputum samples of a patient in the First Affiliated Hospital in Wenzhou, China. FK-2624 was the first strain of *K. pneumoniae* isolated from the patient. After that, multiple swabs for bacterial isolation were taken successively. FK-2624 was chosen to conduct this study as it represented the drug resistance profiles for five of the early isolates (Table S1). Thereafter, FK-2723 was the carbapenem-susceptible strain isolated from the patient immediately before carbapenem treatment was enacted. Subsequently, and after a 14-day treatment with the carbapenem, imipenem, FK-2820 was isolated. Species identification was performed using the Matrix-Assisted Laser Desorption Ionization Time-Of-Flight Mass Spectrometry (MALDITOF MS, Bruker Daltonics, US). After collection, isolates were stored in 30% glycerol at − 80 °C. All of the investigation protocols in this study were approved by the Ethics Committee of the First Affiliated Hospital of Wenzhou Medical University (ethical number 2019–75). Informed consent was waived because this retrospective study focused on the bacterial isolates and had no impact on interventions to the patient.

### Antimicrobial susceptibility testing, detection of resistance genes and virulence factors

The modified carbapenem inactivation method (mCIM) and EDTA synergy test was carried out according to CLSI recommendations for phenotypic screening of carbapenemase producers [22, 23]. Antimicrobial susceptibility test was determined by the agar dilution method and interpreted according to the recommendations in the latest CLSI guidelines, including ceftriaxone (0.015–64 μg/mL), ceftazidime (0.015–64 μg/mL), cefotaxime (0.015–64 μg/mL), imipenem (0.015–16 μg/mL), meropenem (0.015–16 μg/mL), ertapenem (0.0035–16 μg/mL), levofloxacin (0.0035–16 μg/mL), ciprofloxacin (0.0035–16 μg/mL), tobramycin (0.125–256 μg/mL), gentamicin (0.125–256 μg/mL), amikacin (0.25–128 μg/mL) and fosfomycin (0.25–1024 μg/mL). The minimum inhibitory concentrations (MICs) of colistin (0.125–16 μg/mL) and tigecycline (0.125–16 μg/mL) were determined by the broth microdilution method. The latest EUCAST breakpoints (available at http://www.eucast.org/clinical breakpoints/) were used for colistin and tigecycline. All of the antibiotics were purchased from Becton Dickinson (Sparks, MD). *E. coli* ATCC 25922 was used as the quality control strain.

A panel of various, relevant antimicrobial resistance genes were amplified by PCR, and the positive amplicons were further confirmed by DNA sequencing. These resistance genes included those encoding the extended spectrum β-lactamase genes (*bla*_CTX-M_, *bla*_PER_, *bla*_SHV_, *bla*_TEM_ and *bla*_VEB_) [24], carbapenemase genes (*bla*_GES_, *bla*_IMI/NMC-A_, *bla*_SME_, *bla*_KPC_, *bla*_VIM_, *bla*_IMP_, *bla*_NDM_ and *bla*_OXA-48_) [25, 26], AmpC β-lactamase genes (*bla*_CMY_, *bla*_FOX_, *bla*_MOX_, and *bla*_DHA_) [27], plasmid-mediated quinolone resistance (PMQR) genes (*qnrA*, *qnrB*, *qnrC*, *qnrD*, *qnrS*, *aac (6′)-Ib-cr*) [28], *oqxAB* multidrug efflux pump genes [29] and the aminoglycoside resistant genes (*aac(6′)–Ib*, *aac(3′)–Ib*, *rmtB*, *armA*, *APH*, *ANT*, *rmtA*, *rmtB rmtC* and *rmtD*) [30–32]. DNA sequences were characterized by sequence comparisons using BLAST (http://blast.ncbi.nlm.nih.gov/Blast.cgi) [33].

To check for the presence of genes that associated with virulence in *K. pneumoniae* [34–36], virulence profiles were established by PCR, namely capsular serotype K1, K2, K5, K20, K54, K57, hypermucoviscosity phenotype (*magA*), allantoin metabolism (*allS*), regulator of mucoid phenotype A (*rmpA*), iron system capture (*iroN*), adhesion type 3 fimbriae (*mrkD*), iron transport and phosphotransferase function (*kfu*), siderophore (*entB*) and siderophore yersiniabactin (*ybtA*).

### Whole-genome sequencing (WGS)

Genomic DNA of *K. pneumoniae* FK-2624, FK-2723 and FK-2820 were purified using the Bioflux DNA purification kit (Bioflux BSC12S1, Beijing) as recommended by the manufacturer. A total amount of 1 μg DNA per sample was used as input material for the DNA sample preparations. Sequencing libraries were generated using NEBNext® Ultra™ DNA Library Prep Kit for Illumina (NEB E7645S, USA) following manufacturer’s recommendations and index codes were added to attribute sequences to each sample. At last, PCR products were purified (AMPure XP system A63880, Beckman, USA) and libraries were analyzed for size distribution by Agilent2100 Bioanalyzer and quantified using real-time PCR. The genomes were sequenced by Illumina NovaSeq PE150.

Sequence reads for each isolate were assembled individually. All good quality paired reads were assembled using the SOAP denovo [37] into a number of contigs. After assembly, the contigs were scaffolded using MeDuSa [38] and ordered of scaffolds was done using MAUVE version 02.25 [39] using as a reference a complete close genome detected using MagiBlast. Annotated was performed using the National Center for Biotechnology Information’s Prokaryotic Genome Annotation Pipeline [40]. To identify potential antibiotic resistance genes in the genomic sequence of the isolates, sequence alignment of the protein sequences of antibiotic resistance genes in the Center for Genomic Epidemiology (http://www.genomicepidemiology.org/). Virulence genes were identified using the virulence factor database (http://www.mgc.ac.cn/VFs/) and PathogenFinder (https://cge.cbs.dtu.dk/services/PathogenFinder/). Additionally, Kleborate (version 0.3.0) was used to identify resistant and virulent genes with their corresponding subtypes [41, 42]. The nucleotide sequence of the genome of FK-2624, FK-2723 and FK-2820 have been submitted to GenBank with accession no. VIGL00000000, VIGM00000000 and VIGK00000000, respectively. Alignments with the three assembled genomes were done using MAUVE version 02.25 [39]. Sequences that were different between isolates were tested for different GC content, using window-acgt form GLIMMER v3.02 [43]. Putative plasmids were assembled and the resulting plasmid assemblies were annotated with Prokka (v1.12) [44]. Graphical representation was generated using the R packages.

Roary (version 3.12.0) was used to align the assembled genomes and two additional ST660 MLST genomes found in NCBI (GCA_002853165 and GCA_002205995.1) and extract the core genome [45]. The core genome was used to generate a phylogenetical tree using RaxML (version 8.2.9), with a GTR + GAMMA model, and a bootstrapping of 1000 replicates [46].

### MLST determination of genetic relatedness of isolates

Pairwise comparisons method, pulsed-field gel electrophoresis (PFGE) and multi-locus sequencing typing (MLST) were used to establish relatedness between same-patient isolates.

The sequences of the three complete genomes of *K. pneumoniae* isolates were independently used in sequence alignments. The sequence reads were mapped to the reference genomes using the Bowtie2 software, which is good for mapping short sequence reads to medium-sized and large genomes. The alignment of clean data of 3 isolates with reference *K. pneumoniae* MGH 78578 (MDR bacterium isolated from a patient [47], accession number CP000647) was performed with the default settings of programs. Finally, the alignment percentage (supplementary material Table S2) was showed by Bowtie2, revealed a high degree of genetic conservation was observed between the three *K. pneumoniae* strains.

PFGE was carried out on our strains according to the method described previously with minor modification [48]. Genomic DNA was extracted from the *K. pneumoniae* isolates, followed by restriction enzyme *Xba* I (Takara 1093A, Japan) digestion for 2 h. PFGE was performed using a CHEF-Mapper XA PFGE system (Bio-Rad, USA) for 18 h with a switch time 6–36 s. Then DNA fingerprints were revealed by GelRed staining. The banding patterns were visualized by GelDoc XR gel imaging system (Bio-Rad, USA) and cluster analysis of similarity values of the PFGE profiles were finally performed by Quantity One program (BioRad Laboratories, USA). The sequence types (STs) of FK-2624, FK-2723 and FK-2820 were determined by MLST. Seven housekeeping genes (*gapA*, *infB*, *mdh*, *pgi, phoE*, *rpoB*, and *tonB*) were amplified and sequenced according to Diancourt et al. [49]. Alleles and sequence types were assigned by the MLST database (http://www.pasteur.fr/mlst/*Kpneumoniae*.html).

### Outer membrane protein isolation and SDS-PAGE

FK-2624, FK-2723 and FK-2820 were cultured in Muller Hinton broth with shaking overnight at 37 °C. Cells were isolated by centrifugation (4200 rpm for 15 min), the cell pellets were washed with 10 mM Tris-HCl, 5 mM MgCl_2_ (pH 7.3), and the cells then lysed by sonication as described [50]. The cell lysate supernatants were treated with 2% solution of sodium lauroylsarcosinate for 30 min at room temperature, centrifuged 30 min at 17000 rpm and the pellets containing outer membrane proteins (OMPs) were suspended in 10 mM Tris-HCl, 5 mM MgCl_2_ (pH 7.3).

Samples containing the OMPs were mixed with loading buffer (TaKaRa 9173, Japan) and denatured by heating at 100 °C for 3 min. Proteins were then separated by sodium dodecyl sulfate polyacrylamide gel electrophoresis (SDS-PAGE) using 12% polyacrylamide separation gel. Bands corresponding to the major protein species were visualized by staining the gels with 0.2% Coomassie brilliant blue (Solarbio C8430, China) in 10% acetic acid and 45% methanol. *K. pneumoniae* ATCC 13883 (an isolate with known expression of OmpK35 and OmpK36) served as a control strain for OMP profiling.

### Analysis of *ompK35* and *ompk36* genes

The coding sequences of the *ompK35* and *ompK36* genes for the representative isolates were amplified and sequenced using primers listed in Table 2. Amplification was carried out with the following thermal cycling conditions: 5 min at 94 °C and 35 cycles of amplification consisting of 30 s at 94 °C, 30 s at 58 °C, and 1 min at 72 °C, with 10 min at 72 °C for the final extension. DNA fragments were analyzed by electrophoresis in a 1% agarose gel at 120 V for 20 min, stained with GelRed (Biotium 41,003, USA). DNA sequence analysis compared with those of *K. pneumoniae* NTUH-2044 (NCBI accession number AP 006725) was conducted.

**Table 2:** Primers used in this study to amplify porin genes and RT-PCR

*Primer*	*Sequence 5′ → 3′*
**DNA amplification and sequencing**	
*ompK35*-F1	AAGACTACTGGTGGTTATCGCGACCT
*ompK35*-R1	CGACAAAAAGCGCGAAGGTTT
*ompK35*-F2	GTCGAAGCGGCAACCGATTATG
*ompK35*-R2	GCTTCGGCTTTGTCGCCATT
*ompK36*-R1	CCGGTTGAAATAGGGGTAAACAGAC
*ompK36*-F1	CCATTAATCGAGGCTCCTCTTACCA
*ompK36*-F2	GAGTTGCGTTGTAGGTCTGG
*ompK36*-R2	GGCGACACCTACGGTTCTGACAA
**RT-PCR**	
*ompK35*-F	GTCTGGACCACCAATGGC
*ompK35*-R	GATCTGAGTTTCGCCTTTCA
*ompK36*-F	GACCAGACCTACATGCGTGTA
*ompK36*-R	GTATTCCCACTGGCCGTAAC

**Note:***F* Forward (5′) primer. *R* Reverse primer

Isolates were examined by real-time RT-PCR to measure the steady-state levels of the transcripts as an indication of the expression of *ompK35* and *ompK36*. For RNA isolation, *K. pneumoniae* isolates were grown in fresh LB medium at 37 °C overnight. Total RNA was extracted from 3 mL of culture using a RNeasy Mini Kit (Qiagen 74,524, Germany) according to the manufacturer’s instructions. The extracted RNA samples were stored at − 80 °C. Purified RNA was reverse transcribed into cDNA for RT-PCR analysis using a cDNA synthesis kit (TaKaRa 6210A, Japan) according to the manufacturer’s instructions. Gene expression levels were measured with RT-PCR using TB Green® Premix Ex Taq™ (TaKaRa RR420Q, Japan). Primers were designed on the basis of the nucleotide sequence in GenBank (Table 2). Expression of each gene was normalized to that of a housekeeping gene (*rpoB*), the primers used for RT-PCR were *rpoB*-F (5′-AAGGCGAATCCAGCTTGTTCAGC-3′) and *rpoB*-R (5′-TGACGTTGCATGTTCGCACCCATCA-3′). The relative expression of *ompK35* and *ompK36* was then calibrated against the corresponding expression by *K. pneumoniae* ATCC 13883. For quality-controlling strain, relative expression was equal to 1.0. All experiments were performed in triplicate and mean of the Ct values were used for analysis. Gene expression levels were calculated using 2^-*ΔΔ*Ct^ method [51].

### Statistical analysis

The statistical correlation of the expression of *ompk35* and *ompk36* was calculated by Student’s *t* test. SPSS (version 17; IBM, USA, IL) was used for statistical analysis. A *P* value lower than 0.05 was considered statistically significant.

## Supplementary information

**Additional file 1: Table S1.***Klebsiella pneumoniae* isolated during hospitalization. **Table S2.** Genomic DNA comparison between the tested strains, SNPs and (% identity). **Table S3.** MICs of *Pseudomonas aeruginosa.***Table S4.** The allelic profile of ST 660. **Table S5.** Genomic DNA assigned to plasmids.

**Additional file 2: Figure S1.** SDS-PAGE analysis of OMPs from representative strains. M, molecular size marker; M, molecular size marker (TaKaRa 3595Q, Janpan). solid circle, strains not included in this study.

**Additional file 3: Figure S2.** PFGE analysis and MLST of 3 *K. pneumoniae* isolates. Relatedness was analyzed using QualityOne software (Bio-Rad Laboratories, USA). The phylogenetic tree was generated using UPGMA clustering. A genetic similarity index scale is indicated by the vertical line. Solid circle, strains not included in this study

## Data Availability

The datasets used and/or analyzed during the current study are available from the corresponding author on reasonable request.

## Abbreviations

MDR: Multidrug-resistant
MALDI-TOF MS: Matrix-assisted laser desorption/ionization time of flight mass spectrometry
MIC: Minimum inhibitory concentration
PCR: Polymerase chain reaction
WGS: Whole-genome sequencing
SDS-PAGE: Sodium dodecyl sulfate polyacrylamide gel electrophoresis
ESBLs: Extended-spectrum β-Lactamases
CRKP: Carbapenem-resistant *K. pneumoniae*
mCIM: Modified carbapenem inactivation method
PFGE: Pulsed-field gel electrophoresis
MLST: Multi-locus sequencing typing
qRT-PCR: Quantitative reverse transcription polymerase chain reaction

## References

[1] Chung PY. The emerging problems of *Klebsiella pneumoniae* infections: carbapenem resistance and biofilm formation. FEMS Microbiol Lett. 2016;363(20):fnw219. 10.1093/femsle/fnw219 27664057

[2] Heidary M, Nasiri MJ, Dabiri H, Tarashi S (2018). Prevalence of drug-resistant *Klebsiella pneumoniae* in Iran: a review article. Iran J Public Health.

[3] Yigit H, Queenan AM, Anderson GJ, Domenech-Sanchez A, Biddle JW, Steward CD, Alberti S, Bush K, Tenover FC (2001). Novel carbapenem-hydrolyzing beta-lactamase, KPC-1, from a carbapenem-resistant strain of *Klebsiella pneumoniae*. Antimicrob Agents Chemother.

[4] Bialek-Davenet S, Mayer N, Vergalli J, Duprilot M, Brisse S, Pages JM, Nicolas-Chanoine MH (2017). In-vivo loss of carbapenem resistance by extensively drug-resistant *Klebsiella pneumoniae* during treatment via porin expression modification. Sci Rep.

[5] Ye Y, Xu L, Han Y, Chen Z, Liu C, Ming L (2018). Mechanism for carbapenem resistance of clinical *Enterobacteriaceae* isolates. Exp Ther Med.

[6] Bi W, Liu H, Dunstan RA, Li B, Torres VVL, Cao J, Chen L, Wilksch JJ, Strugnell RA, Lithgow T (2017). Extensively drug-resistant *Klebsiella pneumoniae* causing nosocomial bloodstream infections in China: molecular investigation of antibiotic resistance determinants, informing therapy, and clinical outcomes. Front Microbiol.

[7] Hamzaoui Z, Ocampo-Sosa A, Fernandez Martinez M, Landolsi S, Ferjani S, Maamar E, Saidani M, Slim A, Martinez-Martinez L, Boutiba-Ben Boubaker I (2018). Role of association of OmpK35 and OmpK36 alteration and *bla*_ESBL_ and/or *bla*_AmpC_ genes in conferring carbapenem resistance among non-carbapenemase-producing *Klebsiella pneumoniae*. Int J Antimicrob Agents.

[8] Lam MMC, Wyres KL, Judd LM, Wick RR, Jenney A, Brisse S, Holt KE (2018). Tracking key virulence loci encoding aerobactin and salmochelin siderophore synthesis in *Klebsiella pneumoniae*. Genome Med.

[9] Daimon Y, Iwama-Masui C, Tanaka Y, Shiota T, Suzuki T, Miyazaki R, Sakurada H, Lithgow T, Dohmae N, Mori H (2017). The TPR domain of BepA is required for productive interaction with substrate proteins and the beta-barrel assembly machinery complex. Mol Microbiol.

[10] Soltes GR, Martin NR, Park E, Sutterlin HA, Silhavy TJ. Distinctive roles for periplasmic proteases in the maintenance of essential outer membrane protein assembly. J Bacteriol. 2017;199(20):e00418-17. 10.1128/JB.00418-17 PMC5637175 28784813

[11] Nilsson G, Belasco JG, Cohen SN, von Gabain A (1987). Effect of premature termination of translation on mRNA stability depends on the site of ribosome release. Proc Natl Acad Sci U S A.

[12] Bridier A (1918). Exploring foodborne pathogen ecology and antimicrobial resistance in the light of shotgun Metagenomics. Methods Mol Biol.

[13] Mohammad Ali Tabrizi A, Badmasti F, Shahcheraghi F, Azizi O (2018). Outbreak of hypervirulent *Klebsiella pneumoniae* harbouring blaVIM-2 among mechanically-ventilated drug-poisoning patients with high mortality rate in Iran. J Glob Antimicrob Resist.

[14] Smith Moland E, Hanson ND, Herrera VL, Black JA, Lockhart TJ, Hossain A, Johnson JA, Goering RV, Thomson KS (2003). Plasmid-mediated, carbapenem-hydrolysing beta-lactamase, KPC-2, in *Klebsiella pneumoniae* isolates. J Antimicrob Chemother.

[15] Mavroidi A, Katsiari M, Likousi S, Palla E, Roussou Z, Nikolaou C, Maguina A, Platsouka ED (2016). Characterization of ST258 Colistin-resistant, *bla*_KPC_-producing *Klebsiella pneumoniae* in a Greek hospital. Microb Drug Resist.

[16] Ma Y, Xu X, Guo Q, Wang P, Wang W, Wang M. Characterization of *fosA5*, a new plasmid-mediated fosfomycin resistance gene in *Escherichia coli*. Lett Appl Microbiol. 2015;60(3):259–64. 10.1111/lam.12366 25441705

[17] Russo TA, Marr CM. Hypervirulent *Klebsiella pneumoniae*. Clin Microbiol Rev. 2019;32(3):e00001-19. 10.1128/CMR.00001-19 PMC6589860 31092506

[18] Russo TA, Olson R, Fang CT, Stoesser N, Miller M, MacDonald U, Hutson A, Barker JH, La Hoz RM, Johnson JR. Identification of Biomarkers for Differentiation of Hypervirulent *Klebsiella pneumoniae* from Classical *K. pneumoniae*. J Clin Microbiol. 2018;56(9):e00776-18. 10.1128/JCM.00776-18 PMC6113484 29925642

[19] Shen Z, Ding B, Ye M, Wang P, Bi Y, Wu S, Xu X, Guo Q, Wang M. High ceftazidime hydrolysis activity and porin OmpK35 deficiency contribute to the decreased susceptibility to ceftazidime/avibactam in KPC-producing *Klebsiella pneumoniae*. J Antimicrob Chemother. 2017;72(7):1930–6. 10.1093/jac/dkx066 28333323

[20] Wozniak A, Villagra NA, Undabarrena A, Gallardo N, Keller N, Moraga M, Roman JC, Mora GC, Garcia P (2012). Porin alterations present in non-carbapenemase-producing *Enterobacteriaceae* with high and intermediate levels of carbapenem resistance in Chile. J Med Microbiol.

[21] Hao M, Ye M, Shen Z, Hu F, Yang Y, Wu S, Xu X, Zhu S, Qin X, Wang M (2018). Porin deficiency in Carbapenem-resistant *Enterobacter aerogenes* strains. Microb Drug Resist.

[22] CLSI (2019). Performance standard for antimicrobial susceptibility Testing.29th ed. CLSI supplement M100.

[23] Drieux L, Brossier F, Sougakoff W, Jarlier V (2008). Phenotypic detection of extended-spectrum beta-lactamase production in *Enterobacteriaceae*: review and bench guide. Clin Microbiol Infect.

[24] Hijazi SM, Fawzi MA, Ali FM, Abd El Galil KH (2016). Prevalence and characterization of extended-spectrum beta-lactamases producing *Enterobacteriaceae* in healthy children and associated risk factors. Ann Clin Microbiol Antimicrob.

[25] Beltrame A, Sarmati L, Cudillo L, Cerretti R, Picardi A, Anemona L, Fontana C, Andreoni M, Arcese W (2009). A fatal case of invasive fungal sinusitis by *Scopulariopsis acremonium* in a bone marrow transplant recipient. Int J Infect Dis.

[26] Lu H, Zhang X, Zhang Y, Liu H, Xu C, Zhang M, Bi W, Zhou T (2018). First report of multidrug-resistant Escherichia coli isolates co-harbouring *mcr-1* and *bla*_OXA-48_ from clinical patients in China. J Glob Antimicrob Resist.

[27] Liu Z, Zhang J, Rao S, Sun L, Zhang J, Liu R, Zheng G, Ma X, Hou S, Zhuang X (2015). Heptaplex PCR melting curve analysis for rapid detection of plasmid-mediated AmpC beta-lactamase genes. J Microbiol Methods.

[28] Buruk CK, Oztel Ocak H, Bayramoglu G, Aydin F (2016). Investigation of plasmid-mediated quinolone resistance genes in quinolone-resistant *Escherichia coli* and *Klebsiella spp*. isolates from bloodstream infections. Mikrobiyol Bul.

[29] Zhang X, Zhang Y, Wang F, Wang C, Chen L, Liu H, Lu H, Wen H, Zhou T (2018). Unravelling mechanisms of nitrofurantoin resistance and epidemiological characteristics among *Escherichia coli* clinical isolates. Int J Antimicrob Agents.

[30] Jana S, Deb JK (2006). Molecular understanding of aminoglycoside action and resistance. Appl Microbiol Biotechnol.

[31] Lin T, Tang CG, Li QH, Ji J, Ge HY, Zhang XY, Sun HP (2015). Identification of *aac(2′)-I* type b aminoglycoside-modifying enzyme genes in resistant *Acinetobacter baumannii*. Genet Mol Res.

[32] Wangkheimayum J, Paul D, Dhar D, Nepram R, Chetri S, Bhowmik D, Chakravarty A, Bhattacharjee A. Occurrence of acquired 16S rRNA methyltransferase-mediated aminoglycoside resistance in clinical Isolates of *Enterobacteriaceae* within a Tertiary Referral Hospital of Northeast India. Antimicrob Agents Chemother. 2017;61(6)e01037-16. 10.1128/AAC.01037-16 PMC5444189 28320725

[33] Doublet B, Boyd D, Douard G, Praud K, Cloeckaert A, Mulvey MR (2012). Complete nucleotide sequence of the multidrug resistance IncA/C plasmid pR55 from *Klebsiella pneumoniae* isolated in 1969. J Antimicrob Chemother.

[34] Compain F, Babosan A, Brisse S, Genel N, Audo J, Ailloud F, Kassis-Chikhani N, Arlet G, Decre D (2014). Multiplex PCR for detection of seven virulence factors and K1/K2 capsular serotypes of *Klebsiella pneumoniae*. J Clin Microbiol.

[35] Yu WL, Ko WC, Cheng KC, Lee CC, Lai CC, Chuang YC (2008). Comparison of prevalence of virulence factors for *Klebsiella pneumoniae* liver abscesses between isolates with capsular K1/K2 and non-K1/K2 serotypes. Diagn Microbiol Infect Dis.

[36] Zhang S, Yang G, Ye Q, Wu Q, Zhang J, Huang Y (2018). Phenotypic and genotypic characterization of *Klebsiella pneumoniae* isolated from retail foods in China. Front Microbiol.

[37] Li R, Zhu H, Ruan J, Qian W, Fang X, Shi Z, Li Y, Li S, Shan G, Kristiansen K (2010). De novo assembly of human genomes with massively parallel short read sequencing. Genome Res.

[38] Bosi E, Donati B, Galardini M, Brunetti S, Sagot MF, Lio P, Crescenzi P, Fani R, Fondi M (2015). MeDuSa: a multi-draft based scaffolder. Bioinformatics.

[39] Darling AE, Mau B, Perna NT (2010). progressiveMauve: multiple genome alignment with gene gain, loss and rearrangement. PLoS One.

[40] Tatusova T, DiCuccio M, Badretdin A, Chetvernin V, Nawrocki EP, Zaslavsky L, Lomsadze A, Pruitt KD, Borodovsky M, Ostell J (2016). NCBI prokaryotic genome annotation pipeline. Nucleic Acids Res.

[41] Wyres KL, Wick RR, Gorrie C, Jenney A, Follador R, Thomson NR, Holt KE (2016). Identification of *Klebsiella* capsule synthesis loci from whole genome data. Microb Genom.

[42] Lam MMC, Wyres KL, Duchene S, Wick RR, Judd LM, Gan YH, Hoh CH, Archuleta S, Molton JS, Kalimuddin S (2018). Population genomics of hypervirulent *Klebsiella pneumoniae* clonal-group 23 reveals early emergence and rapid global dissemination. Nat Commun.

[43] Delcher AL, Bratke KA, Powers EC, Salzberg SL (2007). Identifying bacterial genes and endosymbiont DNA with Glimmer. Bioinformatics.

[44] Seemann T (2014). Prokka: rapid prokaryotic genome annotation. Bioinformatics.

[45] Page AJ, Cummins CA, Hunt M, Wong VK, Reuter S, Holden MT, Fookes M, Falush D, Keane JA, Parkhill J (2015). Roary: rapid large-scale prokaryote pan genome analysis. Bioinformatics.

[46] Stamatakis A (2014). RAxML version 8: a tool for phylogenetic analysis and post-analysis of large phylogenies. Bioinformatics.

[47] Ogawa W, Li DW, Yu P, Begum A, Mizushima T, Kuroda T, Tsuchiya T (2005). Multidrug resistance in *Klebsiella pneumoniae* MGH78578 and cloning of genes responsible for the resistance. Biol Pharm Bull.

[48] Yan Z, Zhou Y, Du M, Bai Y, Liu B, Gong M, Song H, Tong Y, Liu Y (2019). Prospective investigation of carbapenem-resistant *Klebsiella pneumonia* transmission among the staff, environment and patients in five major intensive care units, Beijing. J Hosp Infect.

[49] Diancourt L, Passet V, Verhoef J, Grimont PA, Brisse S (2005). Multilocus sequence typing of *Klebsiella pneumoniae* nosocomial isolates. J Clin Microbiol.

[50] Martinez-Martinez L, Conejo MC, Pascual A, Hernandez-Alles S, Ballesta S, Ramirez De Arellano-Ramos E, Benedi VJ, Perea EJ (2000). Activities of imipenem and cephalosporins against clonally related strains of *Escherichia coli* hyperproducing chromosomal beta-lactamase and showing altered porin profiles. Antimicrob Agents Chemother.

[51] Sharifi A, Mohammadzadeh A, Zahraei Salehi T, Mahmoodi P (2018). Antibacterial, antibiofilm and antiquorum sensing effects of Thymus daenensis and Satureja hortensis essential oils against *Staphylococcus aureus* isolates. J Appl Microbiol.

