# Supplementary material for: First description of antimicrobial resistance in carbapenem-susceptible Klebsiella pneumoniae after imipenem treatment, driven by outer membrane remodeling
Source: BMC Microbiol. 2020 Jul 20;20:218. doi: 10.1186/s12866-020-01898-1 (PMC7372807; doi:10.1186/s12866-020-01898-1)
Supplement: Supplementary file 1 — Additional file 1: Table S1.Klebsiella pneumoniae isolated during hospitalization. Table S2. Genomic DNA comparison between the tested strains, SNPs and (% identity). Table S3. MICs of Pseudomonas aeruginosa.Table S4. The allelic profile of ST 660. Table S5. Genomic DNA assigned to plasmids. [file 12866_2020_1898_MOESM1_ESM.docx]

**Table S1** *Klebsiella pneumoniae* isolated during hospitalization.

| **Strain number** | **Sample number** | **Sample** |
| --- | --- | --- |
| FK-2624 | 20151120XJC221 | Sputum |
| * | 20151121XJC237 | Sputum |
| * | 20151123XJC241 | Sputum |
| * | 20151126XJC216 | Sputum |
| * | 20151128XJC203 | Sputum |
| * | 20151130XJC205 | Sputum |
| FK-2723 | 20160104XJC213 | Sputum |
| FK-2820 | 20160214XJC203 | Sputum |

**Note:** *, not reserved.

**Table S2** Genomic DNA comparison between the tested strains, SNPs and (% identity).

|  | **FK-2624** | **FK-2723** | **FK-2820** |
| --- | --- | --- | --- |
| **FK-2624** |  | 244 (98.51%) | 410 (98.51%) |
| **FK-2723** | 244 (98.51%) |  | 215 (98.36%) |
| **FK-2820** | 410 (98.51%) | 215 (98.36%) |  |

**Table S3** MICs of *Pseudomonas aeruginosa*

| MIC(μg/mL) ^1)^ | | | | | | | | | |
| --- | --- | --- | --- | --- | --- | --- | --- | --- | --- |
| TZP | ATM | ATM | CAZ | IPM | CIP | LEV | GEN | TOB | AMK |
| 8 | 4 | 2 | 8 | 16 | 0.5 | 1 | 4 | <1 | 8 |

**Note:** ^1)^ TZP=piperacillin/tazobactam; ATM=aztreonam; ATM=cefepime; CAZ=ceftazidime, IPM=imipenem; CIP=ciprofloxacin, LEV=levofloxacin; GEN=gentamicin; TOB=tobramycin; AMK=amikacin.

**Table S4 The allelic profile of ST 660**

| ST | *gapA* | *infB* | *mdh* | *pgi* | *phoE* | *rpoB* | *tonB* |
| --- | --- | --- | --- | --- | --- | --- | --- |
| 660 | 2 | 1 | 2 | 1 | 4 | 1 | 25 |

**Table S5** Genomic DNA assigned to plasmids.

| **Strain** | **Genome assembly (nts)** | **nts > than FK2624** | **plasmid length (nt)** | **plasmid nts > than FK2624** |
| --- | --- | --- | --- | --- |
| FK-2624 | ~5700000 | 0 | ~60000 |  |
| FK-2723 | ~6054000 | ~355000 | ~388000 | 328000 |
| FK-2820 | ~6057000 | ~357000 | ~400000 | 340000 |
