# Supplementary figures and images for: First description of antimicrobial resistance in carbapenem-susceptible Klebsiella pneumoniae after imipenem treatment, driven by outer membrane remodeling
Source: BMC Microbiol. 2020 Jul 20;20:218. doi: 10.1186/s12866-020-01898-1 (PMC7372807; doi:10.1186/s12866-020-01898-1)

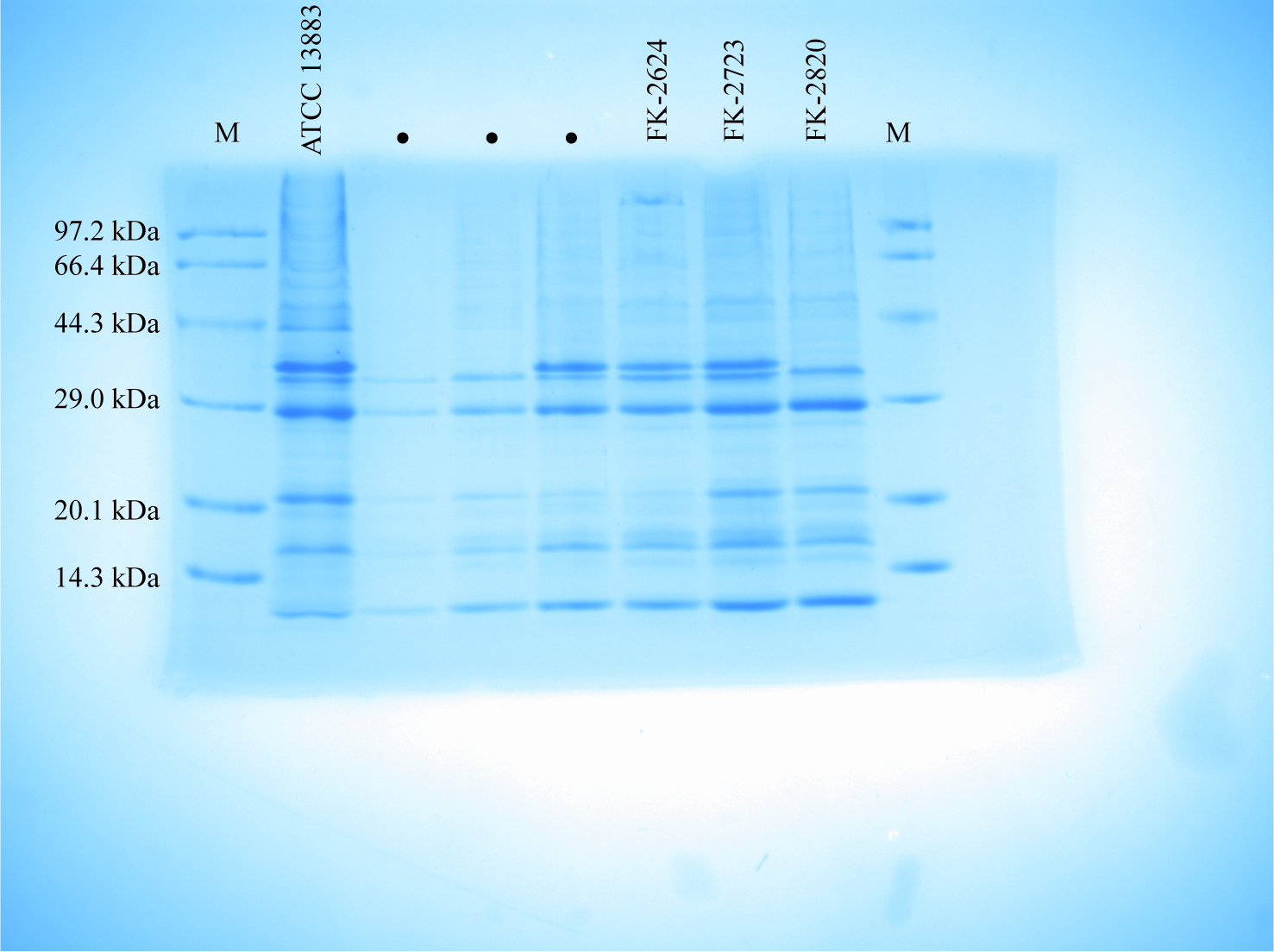

Supplement: Supplementary file 2 — Additional file 2: Figure S1. SDS-PAGE analysis of OMPs from representative strains. M, molecular size marker; M, molecular size marker (TaKaRa 3595Q, Janpan). solid circle, strains not included in this study. [file 12866_2020_1898_MOESM2_ESM.tif]

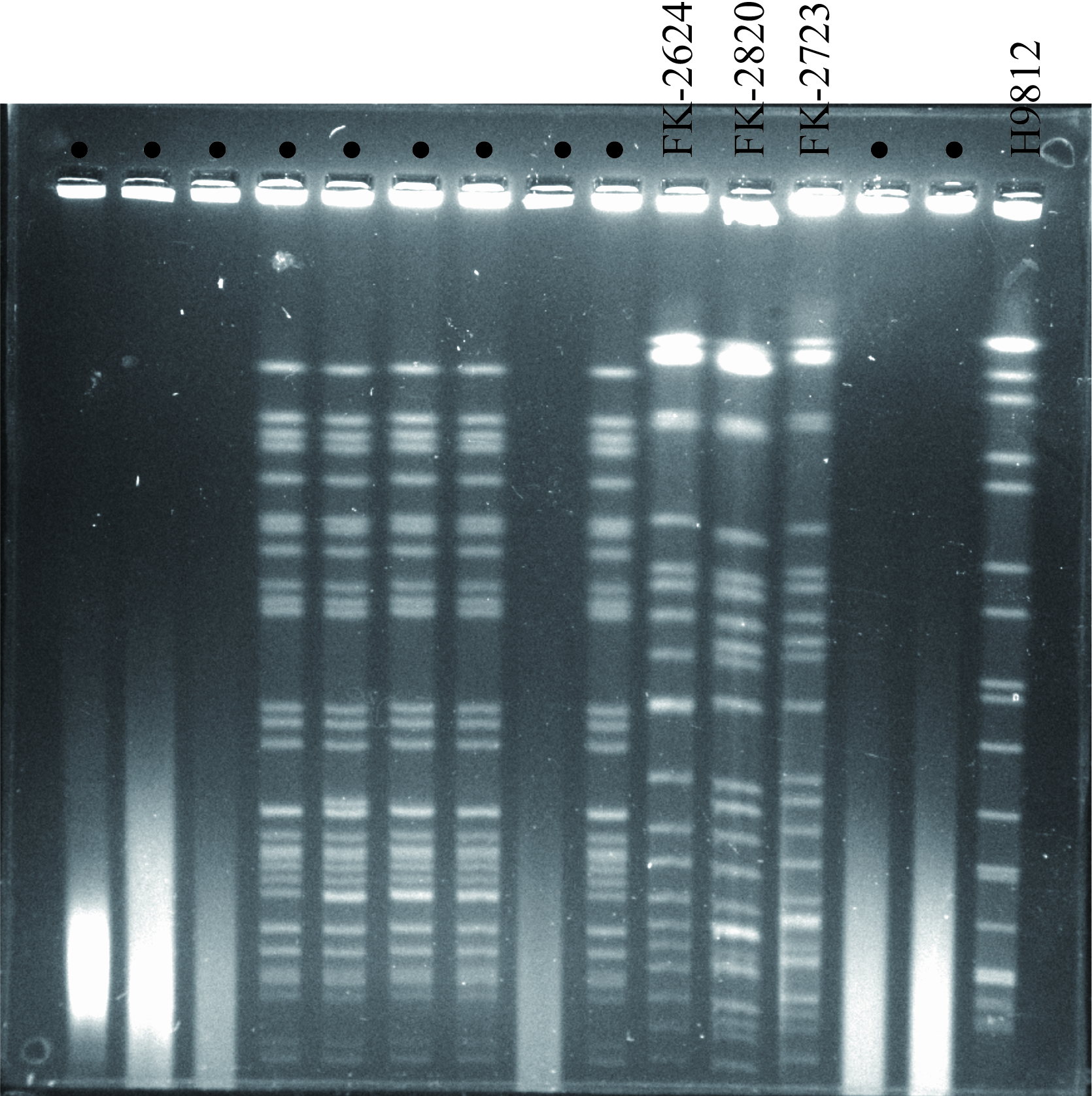

Supplement: Supplementary file 3 — Additional file 3: Figure S2. PFGE analysis and MLST of 3 K. pneumoniae isolates. Relatedness was analyzed using QualityOne software (Bio-Rad Laboratories, USA). The phylogenetic tree was generated using UPGMA clustering. A genetic similarity index scale is indicated by the vertical line. Solid circle, strains not included in this study [file 12866_2020_1898_MOESM3_ESM.tif]
